# Semaglutide Selectively Improves Metabolic and Cognitive Function in 5xFAD Mice

**DOI:** 10.3390/ijms27125311

**Published:** 2026-06-11

**Authors:** Lucy Shahabian, Demos Kynigopoulos, Revekka Papacharalambous, Eleni Ioannou, Sofia Dionysiou, Sylia Christou, Michalis Picolos, Menelaos Pipis, Elena Panayiotou

**Affiliations:** 1 Neuropathology Department, Cyprus Institute of Neurology and Genetics, 2371 Nicosia, Cyprus; lucys@cing.ac.cy (L.S.); kynigopoulosd@cing.ac.cy (D.K.); revekka@cing.ac.cy (R.P.); eleniioann@cing.ac.cy (E.I.); sofiadionysiou20@gmail.com (S.D.); sylia.i.christou@gmail.com (S.C.); menelaosp@cing.ac.cy (M.P.); 2 Cyprus Institute of Neurology and Genetics School, 2371 Nicosia, Cyprus; 3 Alithias Endocrinology Centre, 2324 Nicosia, Cyprus; picolos_endocrine@yahoo.com

**Keywords:** Alzheimer’s disease, metabolic syndrome, insulin resistance, 5xFAD mouse model, neuroinflammation, adipose–brain axis

## Abstract

Alzheimer’s disease (AD) and metabolic syndrome often occur together, sharing characteristics such as insulin resistance, dyslipidemia, and chronic inflammation. Metabolic dysfunction frequently precedes cognitive decline, indicating that early intervention might alter the disease’s progression. We investigated whether the GLP-1 receptor agonist semaglutide (SMGL) influences metabolic impairment and AD pathology in an AD mouse model. Male and female 5xFAD and wild-type (WT) mice on regular (RD) or high-fat diets (HFD) were administered SMGL for 13 weeks. SMGL-treated groups exhibited significant, context-dependent effects. In metabolically challenged 5xFAD HFD mice, treatment led to reduced body weight, improved glucose tolerance, normalized cholesterol levels, and a restored balance of adiponectin and leptin. These improvements were associated with reduced Aβ40 and Aβ42 levels, restored GLP-1 receptor expression, increased synaptophysin and βIII-tubulin levels, and enhanced spatial memory. SMGL also decreased Iba1 and CD68 immunoreactivity in the hippocampus and cortex, reduced macrophage infiltration, and lowered CD36 expression in visceral adipose tissue (VAT), indicating coordinated anti-inflammatory effects. WT RD mice showed minimal metabolic responses and a modest decline in Y-maze performance, suggesting that excessive GLP-1 receptor activation may disrupt neuronal homeostasis when metabolic status is normal. SMGL acts as a context-specific metabolic and neuroprotective agent, offering the greatest benefits under conditions of metabolic dysfunction. These findings in a preclinical model suggest that targeting early metabolic disturbances provides a testable hypothesis for attenuating AD-related neurodegeneration, though further translational studies are required.

## 1. Introduction

Alzheimer’s disease (AD) is a progressive neurodegenerative disorder characterized by chronic neuroinflammation and cognitive decline [1]. It is strongly associated with aging and characterized by the accumulation of extracellular β amyloid plaques and intracellular neurofibrillary tangles composed of hyperphosphorylated tau [2]. Although genetic factors contribute to AD susceptibility, increasing evidence indicates that environmental influences and metabolic disturbances also play a substantial role in disease development and progression [3]. This is especially relevant because amyloid deposition and tau abnormalities typically begin decades before the appearance of mild cognitive symptoms [4,5]. The projected rise in global AD prevalence, together with the escalating financial burden, which is anticipated to exceed 384 billion dollars in 2025 [6], highlights the need to identify practical therapeutic strategies that can modify disease trajectories.

Metabolic syndrome (MetS) is a cluster of interrelated risk factors that significantly increases the likelihood of type 2 diabetes (T2D) [7]. Central obesity, particularly visceral adiposity, is a defining feature of MetS and is associated with persistent low-grade inflammation [8]. Insulin resistance (IR) contributes to the pathogenesis of T2D and is frequently aggravated by excess visceral fat, which promotes lipid accumulation and metabolic stress within the adipose tissue [9,10]. When this signaling is disrupted, neuronal glucose uptake is compromised, contributing to synaptic dysfunction and cognitive impairment [11]. Epidemiological studies link metabolic dysfunction to dementia risk, including a two-fold increase in AD among individuals with T2D, as reported in the Rotterdam Study, and up to a 65 percent increased risk in additional cohorts [12,13]. IR, obesity, and dyslipidemia have been repeatedly associated with increased β amyloid deposition, tau hyperphosphorylation, and accelerated neurodegeneration [14,15]. Inflammatory activation within adipose tissue further contributes to systemic oxidative stress, which in turn influences central immune pathways [16]. The relationship between AD and T2D is increasingly described as bidirectional, with each condition amplifying the pathophysiological processes of the other [17]. In the absence of effective disease-modifying therapies for AD, repurposing existing metabolic interventions represents a realistic and clinically relevant strategy.

In this study, we examined the effects of semaglutide, an FDA-approved metabolic agent, using the 5xFAD mouse model, which develops rapid and aggressive amyloid pathology beginning at approximately two months of age [18]. The primary aim was to determine whether semaglutide could modulate metabolic dysfunction and whether such metabolic correction could influence downstream AD-related pathology. Given the strong epidemiological and mechanistic links between metabolic syndrome (MetS) and AD, we sought to test whether a GLP-1 receptor agonist used in T2D treatment could attenuate key disease features in a transgenic model of amyloid-driven neurodegeneration. We investigated metabolic outcomes under high-fat-diet-induced stress, including changes in body weight, glucose tolerance, lipid handling, and adipokine balance. We further assessed neuroinflammation, focusing on microglial activation and inflammatory signaling in the hippocampus and cortex, and examined AD-related neuropathology, including β-amyloid deposition and synaptic integrity. By comparing semaglutide-treated and untreated 5xFAD mice under regular (RD) and high-fat dietary (HFD) conditions, we aimed to determine whether metabolic intervention was associated with an attenuation of neurodegenerative changes under metabolic stress.

Semaglutide (SMGL) lowers glucose levels by enhancing glucose-dependent insulin secretion, reducing inappropriate glucagon release, slowing gastric emptying, and promoting satiety through central pathways [19,20]. It crosses the blood–brain barrier and engages GLP-1 receptors that are widely expressed in regions implicated in both metabolic regulation and cognitive function, including the hypothalamus and hippocampus [21]. Previous studies suggest that GLP-1 receptor activation may support synaptic plasticity, mitochondrial function, and anti-inflammatory responses while also limiting the accumulation and toxicity of β amyloid [22]. Our study explores whether enhancements in metabolic homeostasis correlate with alterations in AD-related pathology, offering insights into the connection between systemic metabolism and neurodegenerative trajectories.

## 2. Results

### 2.1. Body Weight Changes Throughout Experimentation

**Figure 1:**
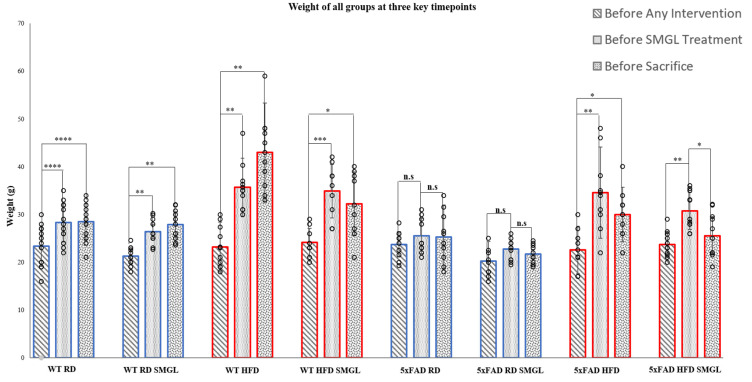
Body weight changes in WT and 5xFAD mice on RD/HFD with/without SMGL treatment. WT RD mice showed a significant increase in their weight after a duration of 5 months, which was stable to the end of the experimental phase. Similarly, WT RD mice treated with SMGL exhibited an increased body weight at 5 months, which remained the same at the endpoint. WT HFD untreated mice showed a significant increase at 5 months, which increased further at the endpoint. The significantly increased weight of the WT HFD SMGL-treated mice at 5 months showed a declining tendency at the endpoint; however, this difference was not significant. Both 5xFAD RD groups, before or after treatment, demonstrated no changes in weight at any stage. 5xFAD HFD mice exhibited a significantly increased body weight at 5 months; however, at the endpoint, their weight was slightly reduced, but not significantly. Finally, the 5xFAD HFD SMGL-treated group showed a significantly increased weight at 5 months. After SMGL administration, their weight was significantly reduced, restoring it to baseline levels [F (2, 85.67) = 71.41]. (WT RD; n = 13, WT RD SMGL; n = 11, WT HFD; n = 11, WT HFD SMGL; n = 9, 5xFAD RD; n = 10, 5xFAD RD SMGL; n = 10, 5xFAD HFD; n = 10, 5xFAD HFD SMGL; n = 11). Mean ± SD. All groups met the normality assumptions (Shapiro–Wilk test; all *p* > 0.05), justifying the use of parametric two-way ANOVA. Two-way ANOVA and Tukey’s post hoc test. * *p* ≤ 0.05, ** *p* ≤ 0.01, *** *p* ≤ 0.001 and **** *p* ≤ 0.0001, n.s = not significant. Circles indicate individual values.

Body weight was recorded at three key time points: 1. baseline, before diet allocation (at 2 months of age); 2. after 5 months on the diet (7 months of age, prior to treatment); and 3. at the end of the 13-week SMGL intervention (9 months of age, before sacrifice) (Figure 1). This effect was observed in both WT and 5xFAD animals, although the overall weight gain was less pronounced in 5xFAD mice, which reached approximately 60% of the gain observed in their WT counterparts. In contrast, animals maintained on a regular diet exhibited stable body weight throughout the same period.

Distinct trends emerged between the groups after the initiation of SMGL treatment. A significant reduction in body weight was observed in both WT-HFD+SMGL and 5xFAD-HFD+SMGL groups relative to their untreated HFD controls (*p* < 0.05). The strongest reduction was recorded in 5xFAD-HFD+SMGL mice, which displayed the greatest percentage decrease in body weight over the treatment period. By comparison, SMGL administration did not significantly alter body weight in RD-fed animals of either genotype.

Overall, HFD caused marked weight gain in both genotypes, whereas SMGL treatment attenuated this effect specifically in HFD-fed groups, with the greatest reduction observed in 5xFAD mice. Body weight remained unchanged in RD-fed animals following SMGL administration. Monthly weight recordings can be found in Figure S1.

### 2.2. Semaglutide Treatment Improved Glucose Levels in Metabolically Challenged 5xFAD Mice

**Figure 2:**
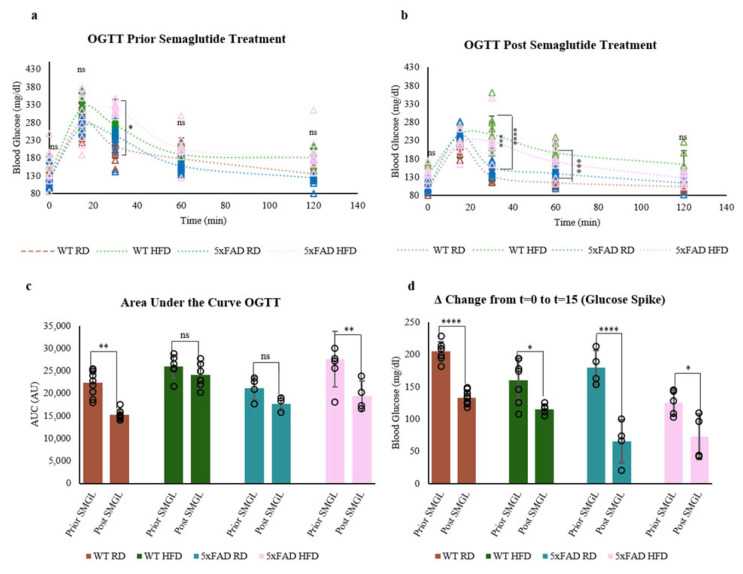
OGTT performed in HFD-fed and RD-fed mice before and after the commencement of SMGL treatment. Glucose levels during OGTT. Fasting glucose levels were measured at 15, 30, 60, and 120 min after oral glucose administration before (**a**) and after (**b**) SMGL treatment. (**a**) 5xFAD HFD showed a significant increase at 30 min [F (3, 100) = 7.987]. (WT RD; n = 11, WT HFD; n = 11, 5xFAD RD; n = 11, 5xFAD HFD; n = 11). (**b**) After treatment, the entire glucose curve was significantly reduced [F (3, 90) = 27.72]. (WT RD; n = 8, WT HFD; n = 8, 5xFAD RD; n = 8, 5xFAD HFD; n = 8). (**c**) The glucose area under the curve (AUC) during OGTT was significantly reduced after SMGL administration in WT RD and 5xFAD HFD. However, no significant changes were observed in the other groups. (**d**) The delta change in the initial spike in blood glucose 15 min after oral glucose administration was significantly reduced after SMGL administration in all groups. Mean ± SD. All groups met the normality assumptions (Shapiro–Wilk test; all *p* > 0.05). One-way ANOVA and Tukey’s post hoc test. * *p* ≤ 0.05, ** *p* ≤ 0.01, *** *p* ≤ 0.001 and **** *p* ≤ 0.0001, ns = not significant. Circles indicate individual values.

To assess the effects of diet and SMGL treatment on glucose homeostasis, oral glucose tolerance tests (OGTTs) were performed in all groups before and after the 13-week intervention (Figure 2a–d). Before treatment, HFD-fed mice displayed impaired glucose tolerance compared to those on RD, as shown by elevated glucose levels at early time points and a delayed return to baseline (*p* < 0.01) (Figure 2a). WT-HFD animals reached a peak of approximately 320 mg/dL at 15–30 min following glucose administration, whereas WT-RD mice peaked at approximately 280 mg/dL and recovered toward baseline by 60 min, indicating normal glucose clearance. Similarly, 5xFAD-HFD mice exhibited a pronounced rise in glucose at 30 min, confirming diet-induced metabolic dysfunction (Figure 2a).

Following SMGL treatment, a distinct improvement in glucose handling was observed. WT-HFD+SMGL animals displayed a reduced 15 min glucose peak (~244 mg/dL, ~24% decrease from baseline) compared with their pretreatment values, whereas 5xFAD-HFD+SMGL mice demonstrated a more efficient decline in glucose levels over time (*p* < 0.05) (Figure 2b). The area under the curve (AUC) analysis (Figure 2c) confirmed these findings, showing a significant reduction in AUC values in the 5xFAD-HFD+SMGL and WT-RD+SMGL groups (*p* < 0.05). The other groups showed a downward trend in AUC post-treatment, although these differences did not reach statistical significance post-treatment.

Overall, SMGL treatment enhanced glucose tolerance in metabolically challenged groups, with the strongest improvement observed in 5xFAD-HFD mice. By comparison, the glucose profiles of WT-RD animals remained stable. Individual OGTT values are shown in Figure S2.

### 2.3. Semaglutide Improved the Spatial Memory in Metabolically Challenged 5xFAD Mice

**Figure 3:**
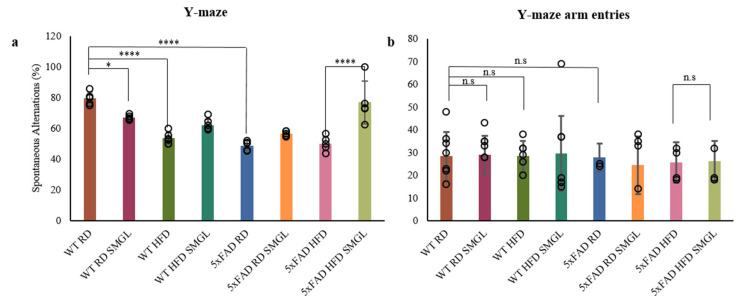
Cognitive impairment assessment using the Y-maze spontaneous alternation test. All groups of mice underwent spontaneous alternation behavioral testing using a Y-maze. (**a**) WT HFD and 5xFAD RD showed a significant reduction in cognitive function compared to WT RD. 5xFAD HFD mice after SMGL administration exhibited increased cognitive function compared to 5xFAD HFD [F (7, 30) = 16.14]. (WT RD; n = 5, WT RD SMGL; n = 5, WT HFD; n = 6, WT HFD SMGL; n = 7, 5xFAD RD; n = 5, 5xFAD RD SMGL; n = 5, 5xFAD HFD; n = 5, 5xFAD HFD SMGL; n = 5). (**b**) Graphical representation of the number of mouse entries in the Y-maze arms. The number of Y-maze entries did not change significantly in any group, either with or without drug administration. This ensured proper motor function and indicated that all animals performed correctly within the given time, irrespective of the outcome [F (7, 55) = 0.2091]. WT RD, n = 14; WT RD OZ, n = 13; WT HFD, n = 10; WT HFD SMGL, n = 10; 5xFAD RD, n = 5; 5xFAD RD SMGL, n = 5; 5xFAD HFD, n = 7; 5xFAD HFD SMGL. All groups met the normality assumptions (Shapiro–Wilk test; all *p* > 0.05), justifying the use of parametric one-way ANOVA. Mean ± SD. One-way ANOVA and Tukey’s post hoc test. * *p* ≤ 0.05, **** *p* ≤ 0.0001 and n.s = not significant.

Spatial working memory was evaluated using a spontaneous alternation Y-maze task (Figure 3). 5xFAD mice displayed significantly lower percentages of spontaneous alternation compared to their WT counterparts (*p* < 0.01). HFD further reduced alternation scores in both genotypes compared to the RD controls (*p* < 0.05). By contrast, SMGL-treated WT-RD mice demonstrated a significant decline in alternation performance (*p* < 0.05) relative to untreated WT-RD controls. No significant change was detected in the WT-HFD or 5xFAD-ND groups after treatment.

Overall, the data suggest that SMGL improved spatial memory in 5xFAD-HFD mice exhibiting metabolic and cognitive deficits while producing a slight decline in task performance in WT-RD mice with preserved cognitive function.

### 2.4. Semaglutide Modulated Amyloid Pathology in Metabolically Challenged 5xFAD Mice

**Figure 4:**
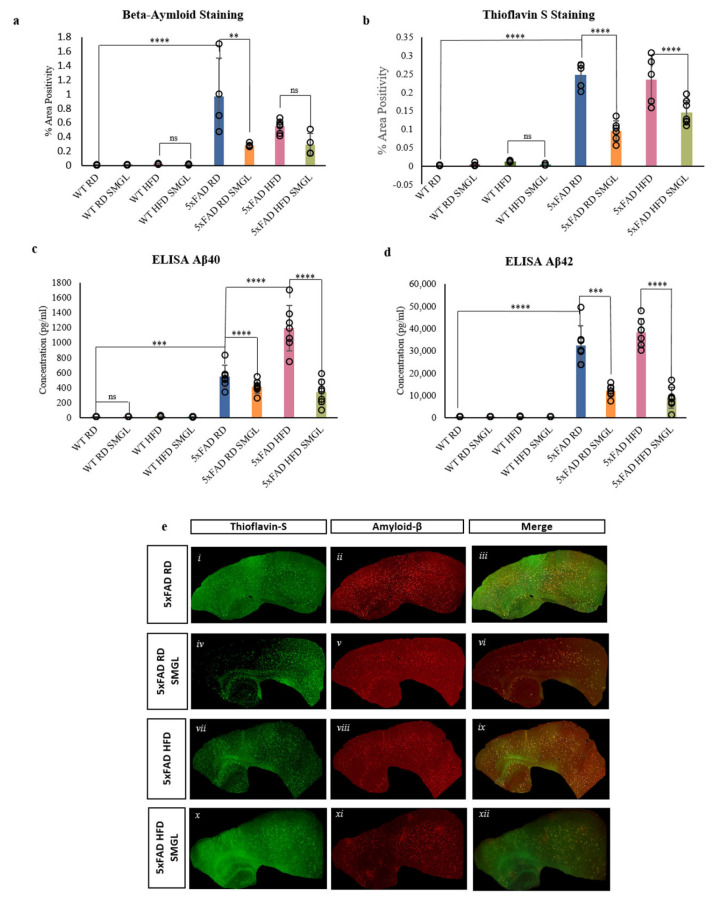
Amyloid load decrease and cognitive impairment amelioration in SMGL-treated animals. Sagittal brain sections from WT RD, WT HFD, 5xFAD RD, and 5xFAD HFD mice, either untreated or treated with SMGL, were co-stained with thioflavin-S (green; excitation λ: 430, emission λ: 550) and an anti-amyloid-β antibody (red; excitation λ: 555, emission λ: 580). (**a**) β-amyloid staining revealed increased expression in 5xFAD RD mice compared to WT controls. After SMGL treatment, β-amyloid levels were significantly reduced. 5xFAD HFD mice also exhibited increased levels of β-amyloid, but no changes were observed after treatment [F (7, 26) = 13.44]. (WT RD; n = 7, WT RD SMGL; n = 4, WT HFD; n = 4, WT HFD SMGL; n = 4, 5xFAD RD; n = 4, 5xFAD RD SMGL; n = 4, 5xFAD HFD; n = 6, 5xFAD HFD SMGL; n = 4). (**b**) Similarly, thioflavin-S staining showed substantial plaque deposition in 5xFAD RD when compared to WT RD, which was significantly reduced after treatment. 5xFAD HFD mice also exhibited increased levels of thioflavin-S-positive plaques that were reduced after treatment [F (7, 32) = 49.96]. (WT RD; n = 5, WT RD SMGL; n = 5, WT HFD; n = 5, WT HFD SMGL; n = 4, 5xFAD RD; n = 5, 5xFAD RD SMGL; n = 6, 5xFAD HFD; n = 5, 5xFAD HFD SMGL; n = 7). (**c**,**d**) Enzyme-linked immunosorbent assay of the two most prominent amyloidogenic species in AD, namely, Aβ40 and Aβ42, respectively. In both cases, the 5xFAD RD and 5xFAD HFD groups demonstrated significantly higher levels of β-amyloid, which were reduced after SMGL treatment: Aβ40—[F (7, 36) = 37.91]; Aβ42—[F (7, 48) = 55.68]. (WT RD; n = 8, WT RD SMGL; n = 8, WT HFD; n = 5, WT HFD SMGL; n = 8, 5xFAD RD; n = 7, 5xFAD RD SMGL; n = 7, 5xFAD HFD; n = 7, 5xFAD HFD SMGL; n = 7). All groups met the normality assumptions (Shapiro–Wilk test; all *p* > 0.05), justifying the use of parametric one-way ANOVA. Mean ± SD. One-way ANOVA and Tukey’s post hoc test. ** *p* ≤ 0.01, *** *p* ≤ 0.001 and **** *p* ≤ 0.0001, ns = not significant. (**e**) Representative sections of the 5xFAD-treated and untreated groups indicating thioflavin-S and β-amyloid staining in the cortex and hippocampus (composite figures from 50× images). Thioflavin-S staining in 5xFAD-treated groups (**iv**,**x**) showed significantly reduced amounts of plaques compared to untreated groups (**i**,**vii**). Similarly, amyloid-b staining in 5xFAD-treated groups (**v**,**xi**) showed significantly reduced amounts of plaques compared to untreated groups (**ii**,**viii**). (**iii**,**vi**,**ix**,**xii**) Co-staining of thioflavin-S and beta-amyloid staining in 5xFAD animals.

Amyloid burden was evaluated histologically and biochemically to determine the effects of SMGL on amyloid pathology in 5xFAD mice (Figure 4a–e). Both β-amyloid immunoreactivity and thioflavin-S-positive plaque deposition (Figure 4a and Figure 4b, respectively) were markedly increased in 5xFAD mice compared to WT controls (*p* < 0.0001). HFD further intensified amyloid accumulation, producing denser plaque staining in the cortex and hippocampus relative to RD conditions (Figure 4e) (*p* < 0.05).

Following SMGL treatment, a significant reduction in both β-amyloid immunostaining and thioflavin-S plaque burden was observed in the 5xFAD-RD and 5xFAD-HFD groups (*p* < 0.05) (Figure 4a and Figure 4b, respectively). These histological findings were consistent with biochemical analyses of soluble amyloid-β species (Figure 4c,d). ELISA quantification showed elevated Aβ40 and Aβ42 concentrations in all 5xFAD groups compared with those in WT animals (*p* < 0.0001), with higher levels detected under HFD conditions. SMGL treatment significantly reduced both Aβ40 and Aβ42 levels in 5xFAD mice on either diet (*p* < 0.05), with the strongest effect observed in 5xFAD-HFD+SMGL animals, in the immunoassay data (Figure 4c,d). No significantly detectable Aβ signals were found in WT samples across all groups; however, higher signals were recorded in the WT-HFD group (Figure S3).

Overall, these results demonstrate that SMGL treatment attenuates amyloid pathology in 5xFAD mice by reducing both plaque deposition and soluble Aβ40/Aβ42 species, with the most pronounced reduction observed under HFD conditions.

### 2.5. Semaglutide Restored Adipokine Balance in 5xFAD Mice

**Figure 5:**
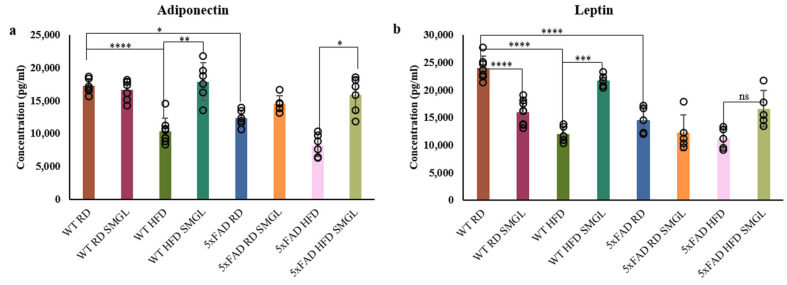
Brain immunoassays of adipokines. Enzyme-linked immunosorbent assay of brain adiponectin (**a**) and leptin (**b**) levels. (**a**) WT HFD, 5xFAD RD, and 5xFAD HFD groups showed significantly reduced levels of adiponectin when compared to the WT RD group. After treatment, adiponectin levels significantly increased, restoring them to WT levels [F (7, 30) = 14.32]. (WT RD; n = 6, WT RD SMGL; n = 7, WT HFD; n = 8, WT HFD SMGL; n = 6, 5xFAD RD; n = 6, 5xFAD RD SMGL; n = 6, 5xFAD HFD; n = 6, 5xFAD HFD SMGL; n = 6). (**b**) Leptin levels in WT-HFD mice were significantly decreased compared to WT RD. The reduced levels of WT HFD were significantly increased after treatment. On the contrary, no significant changes were observed after treatment to the reduced levels of 5xFAD RD and 5xFAD HFD [F (7, 31) = 17.95]. (WT RD; n = 7, WT RD SMGL; n = 8, WT HFD; n = 5, WT HFD SMGL; n = 5, 5xFAD RD; n = 5, 5xFAD RD SMGL; n = 5, 5xFAD HFD; n = 5, 5xFAD HFD SMGL; n = 5). All groups met normality assumptions (Shapiro–Wilk test; all *p* > 0.05), justifying the use of parametric one-way ANOVA. Mean ± SD. One-way ANOVA and Tukey’s post hoc test. * *p* ≤ 0.05, ** *p* ≤ 0.01, *** *p* ≤ 0.001 and **** *p* ≤ 0.0001, ns = not significant. Circles indicate individual values.

To assess the impact of diet, genotype, and SMGL treatment on brain adipokine levels, ELISA assays were performed for adiponectin and leptin (Figure 5). Adiponectin expression was significantly decreased in HFD mice of both genotypes (*p* < 0.01). Among the untreated groups, 5xFAD and WT mice on HFD displayed the lowest adiponectin concentrations compared to their respective RD controls. Following SMGL treatment, adiponectin levels increased significantly in WT-HFD and 5xFAD-HFD groups (*p* < 0.05), whereas WT animals showed no significant response (Figure 5a).

Leptin levels follow a similar pattern, where HFD feeding led to reduced leptin levels in WT-HFD, 5xFAD-RD and 5xFAD-HFD animals compared to their WT-RD controls. SMGL treatment elevated leptin concentrations in HFD-treated WT mice specifically, as well as an increasing trend in 5xFAD HFD post-SMGL treatment, although this was not significant. WT groups showed minimal change (Figure 5b).

Overall, these findings demonstrate that SMGL restored a more favorable adipokine balance in 5xFAD mice by increasing brain adiponectin, particularly under HFD conditions, whereas its effects in WT animals were limited.

### 2.6. Semaglutide Restored Glp-1R Expression in 5xFAD Mice

**Figure 6:**
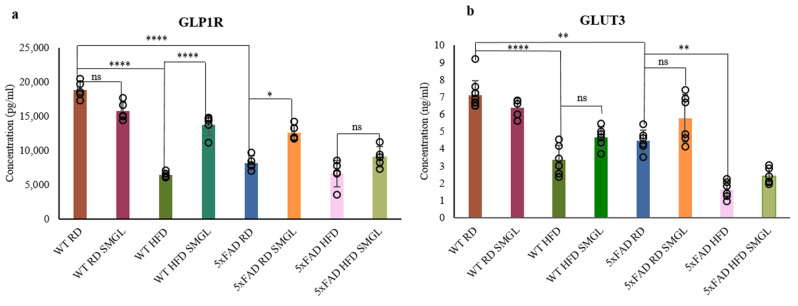
Brain immunoassays of receptors. Enzyme-linked immunosorbent assay (ELISA) of GLP1R (**a**) and GLUT3 (**b**) receptor levels in the brain. (**a**) The expression levels of GLP1R remained unaffected in the WT RD group after treatment. However, the reduced levels in WT HFD and 5xFAD RD were significantly increased after treatment [F (7, 23) = 37.08]. (WT RD; n = 5, WT RD SMGL; n = 5, WT HFD; n = 5, WT HFD SMGL; n = 5, 5xFAD RD; n = 5, 5xFAD RD SMGL; n = 5, 5xFAD HFD; n = 5, 5xFAD HFD SMGL; n = 5). (**b**) GLUT3 levels were significantly reduced in WT HFD and 5xFAD RD when compared to WT RD. Additionally, 5xFAD HFD exhibited further reduced levels when compared to the 5xFAD RD, and SMGL treatment did not significantly alter the expression of GLUT3 in any WT or 5xFAD groups [F (7, 28) = 29.71]. (WT RD; n = 10, WT RD SMGL; n = 5, WT HFD; n = 6, WT HFD SMGL; n = 6, 5xFAD RD; n = 6, 5xFAD RD SMGL; n = 6, 5xFAD HFD; n = 6, 5xFAD HFD SMGL; n = 6). All groups met normality assumptions (Shapiro–Wilk test; all *p* > 0.05), justifying the use of parametric one-way ANOVA. Mean ± SD. One-way ANOVA and Tukey’s post hoc test. * *p* ≤ 0.05, ** *p* ≤ 0.01, and **** *p* ≤ 0.0001, ns = not significant. Circles indicate individual values.

To examine how diet, genotype, and SMGL treatment affected central receptor and transporter levels, GLP-1 receptor (GLP-1R) and glucose transporter 3 (GLUT3) expression were quantified in brain homogenates (Figure 6). GLP-1R expression was significantly lower in 5xFAD mice compared with WT-RD controls (*p* < 0.0001). HFD feeding further suppressed GLP-1R levels in both genotypes (*p* < 0.05). Following SMGL administration, GLP-1R expression was restored in 5xFAD-RD and 5xFAD-HFD groups (*p* < 0.05), approaching levels comparable to WT-RD. In contrast, SMGL treatment did not significantly alter GLP-1R expression in WT animals maintained on either diet (Figure 6a).

GLUT3 expression exhibited a similar but less responsive pattern. Baseline GLUT3 levels were reduced in 5xFAD mice compared to WT mice (*p* < 0.01) and were further decreased by HFD (*p* < 0.0001). SMGL treatment did not significantly modify GLUT3 expression in any group (Figure 6b).

Overall, SMGL treatment restored GLP-1R expression in 5xFAD mice under both dietary conditions, whereas GLUT3 expression remained unchanged, indicating selective receptor sensitivity to treatment.

### 2.7. Semaglutide Improved Neuronal Integrity and Reduced Neuroinflammation in 5xFAD Mice

**Figure 7:**
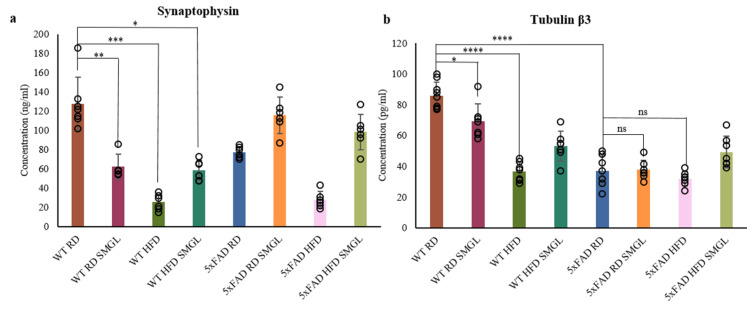
Brain immunoassays of neural markers. Expression levels of enzyme-linked immunosorbent assay of brain synaptophysin [F (7, 21) = 11.33] (**a**) and tubulin-β3 [F (7, 36) = 25.72] (**b**). A significant reduction in synaptophysin and tubulin-β3 was observed in all groups when compared with the WT RD group. (WT RD; n = 7, WT RD SMGL; n = 5, WT HFD; n = 6, WT HFD SMGL; n = 6, 5xFAD RD; n = 6, 5xFAD RD SMGL; n = 6, 5xFAD HFD; n = 6, 5xFAD HFD SMGL; n = 6). All groups met the normality assumptions (Shapiro–Wilk test; all *p* > 0.05), justifying the use of parametric one-way ANOVA. Mean ± SD. One-way ANOVA and Tukey’s post hoc test. * *p* ≤ 0.05, ** *p* ≤ 0.01, *** *p* ≤ 0.001 and **** *p* ≤ 0.0001, ns = not significant.

**Figure 8:**
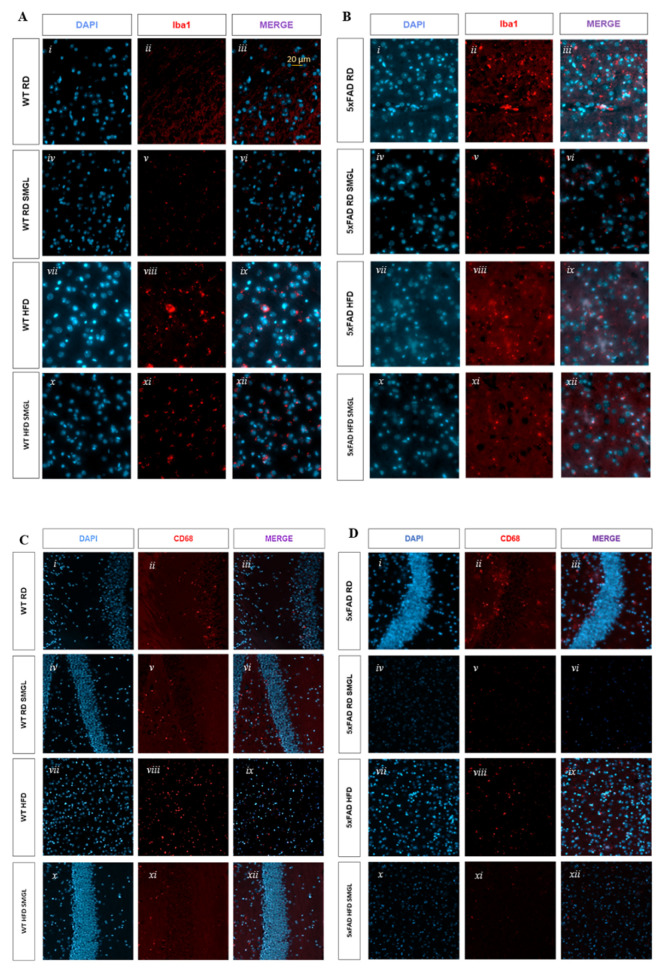
Microglial activation and phagocytosis assessed by Iba1 and CD68 immunofluorescence in WT and 5xFAD brain tissue. Representative cortical images of WT (**A**,**C**) and 5xFAD (**B**,**D**) mice maintained on an RD or HFD, with or without SMGL treatment. Panels (**A**,**B**) show DAPI (blue), Iba1 (red), and merged images. Panels (**C**,**D**) show DAPI (blue), CD68 (red), and merged images. (**A**) WT RD control mice: (**i**) DAPI, (**ii**) Iba1, and (**iii**) merged image; WT RD+SMGL mice: (**iv**) DAPI, (**v**) Iba1, and (**vi**) merged image; WT HFD mice: (**vii**) DAPI, (**viii**) Iba1, and (**ix**) merged image; WT HFD+SMGL mice: (**x**) DAPI, (**xi**) Iba1, and (**xii**) merged image. WT RD mice exhibited minimal Iba1 immunoreactivity, whereas increased staining was observed in WT HFD mice and appeared reduced following SMGL Treatment. (**B**) 5xFAD RD mice: (**i**) DAPI, (**ii**) Iba1, and (**iii**) merged image; 5xFAD RD+SMGL mice: (**iv**) DAPI, (**v**) Iba1, and (**vi**) merged image; 5xFAD HFD mice: (**vii**) DAPI, (**viii**) Iba1, and (**ix**) merged image; 5xFAD HFD+SMGL mice: (**x**) DAPI, (**xi**) Iba1, and (**xii**) merged image. Increased Iba1 immunoreactivity was observed in 5xFAD mice and appeared reduced following SMGL treatment under both dietary conditions. (**C**) WT RD control mice: (**i**) DAPI, (**ii**) CD68, and (**iii**) merged image; WT RD+SMGL mice: (**iv**) DAPI, (**v**) CD68, and (**vi**) merged image; WT HFD mice: (**vii**) DAPI, (**viii**) CD68, and (**ix**) merged image; WT HFD+SMGL mice: (**x**) DAPI, (**xi**) CD68, and (**xii**) merged image. CD68 immunoreactivity was minimal in WT RD mice but increased in WT HFD mice and appeared reduced following SMGL treatment. (**D**) 5xFAD RD control mice: (**i**) DAPI, (**ii**) CD68, and (**iii**) merged image; 5xFAD RD+SMGL mice: (**iv**) DAPI, (**v**) CD68, and (**vi**) merged image; 5xFAD HFD mice: (**vii**) DAPI, (**viii**) CD68, and (**ix**) merged image; 5xFAD HFD+SMGL mice: (**x**) DAPI, (**xi**) CD68, and (**xii**) merged image. Increased CD68 immunoreactivity was observed in 5xFAD mice and appeared reduced following SMGL treatment under both dietary conditions.

Synaptic and neuronal integrity were evaluated by quantifying synaptophysin (SYP) and βIII-tubulin (Tubb3) levels (Figure 7), whereas neuroinflammatory changes were assessed by immunostaining for the microglial marker Iba1 and macrophage/activation marker CD68 (Figure 8).

Both SYP and Tubb3 levels were significantly reduced in 5xFAD mice compared to those in WT-RD controls (*p* < 0.05), reflecting synaptic loss and neuronal impairment typical of the 5xFAD phenotype. HFD further decreased the expression of these genes in both genotypes (*p* < 0.05). Following SMGL treatment, SYP and Tubb3 levels were significantly increased in 5xFAD-HFD mice (*p* < 0.05), partially restoring them to WT baseline values. No significant changes were detected in WT groups after treatment (Figure 7a and Figure 7b, respectively).

Iba1 and CD68 immunoreactivity were examined in the cortex and the hippocampus to assess microglial activation (Figure 8). In the untreated groups, both markers showed stronger labeling in HFD animals of either genotype than in RD controls (*p* < 0.01), with the most pronounced signal in 5xFAD-HFD mice. Elevated Iba1 and CD68 expression was also observed in 5xFAD-RD mice relative to WT-RD mice (*p* < 0.05), indicating baseline neuroinflammatory activity associated with their genotype. SMGL treatment markedly reduced Iba1 and CD68 staining intensity in both 5xFAD-RD and 5xFAD-HFD brains (*p* < 0.05), and to a lesser extent in WT-HFD animals, whereas WT-RD groups remained unchanged (Figure 8A and Figure 8B, respectively).

Overall, these findings demonstrate that SMGL treatment restores neuronal and synaptic protein expression and attenuates microglial activation and macrophage-associated inflammation in 5xFAD mice.

### 2.8. Semaglutide Normalized Total and LDL Cholesterol in Metabolically Challenged 5xFAD Mice

**Figure 9:**
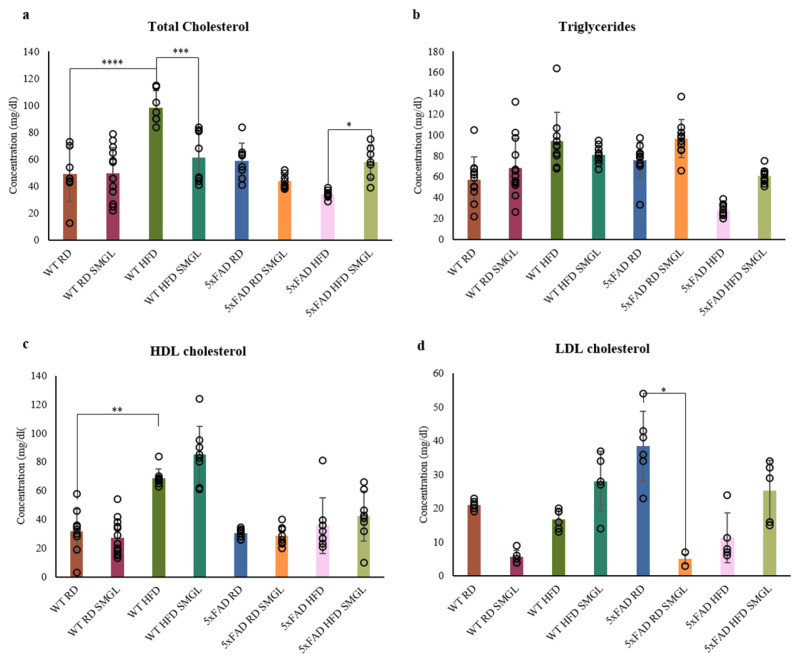
Lipid metabolism assessed by cholesterol, triglycerides, HDL and LDL in SMGL-treated WT and 5xFAD mice. (**a**) Serum samples revealed increased levels of total cholesterol in the WT HFD group compared to the WT RD group. After SMGL treatment, the total cholesterol levels in WT HFD mice were significantly reduced. By contrast, the reduced levels in the 5xFAD group were significantly increased, restoring them to normal levels [F (7, 47) = 9.3]. (WT RD; n = 7, WT RD SMGL; n = 13, WT HFD; n = 7, WT HFD SMGL; n = 8, 5xFAD RD; n = 8, 5xFAD RD SMGL; n = 8, 5xFAD HFD; n = 7, 5xFAD HFD SMGL; n = 7). (**b**) No significant changes were observed in the triglyceride levels between the groups [F (7, 43) = 4.386]. (WT RD; n = 11, WT RD SMGL; n = 13, WT HFD; n = 10, WT HFD SMGL; n = 10, 5xFAD RD; n = 10, 5xFAD RD SMGL; n = 10, 5xFAD HFD; n = 10, 5xFAD HFD SMGL; n = 10). (**c**) HDL levels in the WT HFD group were significantly elevated compared to those in the WT RD group. After treatment, an even greater tendency was observed [F (7, 40) = 8.937]. (WT RD; n = 9, WT RD SMGL; n = 13, WT HFD; n = 9, WT HFD SMGL; n = 8, 5xFAD RD; n = 8, 5xFAD RD SMGL; n = 8, 5xFAD HFD; n = 8, 5xFAD HFD SMGL; n = 8). (**d**) The LDL cholesterol levels in the 5xFAD RD group were higher than in those of their WT RD littermates. After treatment, the LDL levels were significantly reduced [F (7, 22) = 6.283]. (WT RD; n = 6, WT RD SMGL; n = 5, WT HFD; n = 6, WT HFD SMGL; n = 5, 5xFAD RD; n = 6, 5xFAD RD SMGL; n = 6, 5xFAD HFD; n = 5, 5xFAD HFD SMGL; n = 5). All groups met the normality assumptions (Shapiro–Wilk test; all *p* > 0.05), justifying the use of parametric one-way ANOVA. Mean ± SD. One-way ANOVA and Tukey’s post hoc test. * *p* ≤ 0.05, ** *p* ≤ 0.01, *** *p* ≤ 0.001 and **** *p* ≤ 0.0001.

Serum lipid analysis revealed diet- and genotype-dependent alterations that were modulated by SMGL treatment (Figure 9a–d). HFD resulted in a pronounced elevation in total cholesterol levels in WT-HFD mice relative to their RD controls (*p* < 0.0001) (Figure 9a). Notably, 5xFAD mice also exhibited slightly higher baseline cholesterol values than WT-RD animals, consistent with the dyslipidemic profile previously described for this genotype [23].

Following treatment, SMGL significantly reduced total cholesterol in WT-HFD mice (*p* < 0.05), whereas no comparable effect was observed in 5xFAD-HFD animals. Triglyceride concentrations remained largely unchanged among all groups (Figure 9b). HDL-cholesterol levels increased in WT-HFD mice (*p* < 0.01) compared with their RD-fed counterparts and were not modified by treatment (Figure 9c). By contrast, LDL cholesterol was significantly decreased in 5xFAD-RD+SMGL mice relative to untreated 5xFAD-RD controls (*p* < 0.05), whereas WT mice showed no differences (Figure 9d).

Overall, SMGL treatment mitigated cholesterol discrepancies selectively in 5xFAD-HFD mice, improving their lipid profiles, and produced minimal changes in the WT group.

### 2.9. Semaglutide Treatment Attenuated Diet-Induced Adipocyte Hypertrophy in Both WT and 5xFAD Mice

**Figure 10:**
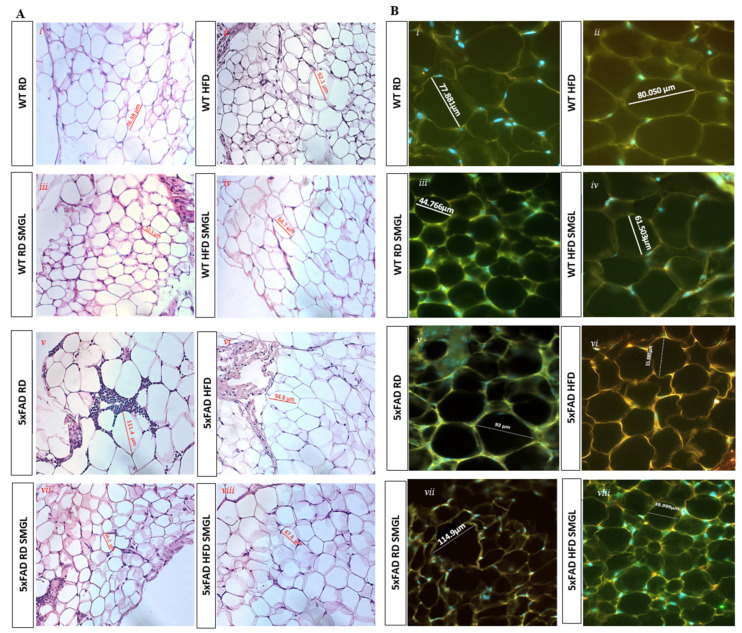
Morphological and inflammatory profiling of VAT using H&E and CD36/F4/80 staining with adipocyte diameter measurements. (**A**) Hematoxylin and eosin staining of visceral adipose tissue at 200× magnification. Representative sections from all groups are shown, with the distance between individual adipocytes indicated. (**A**(**i**)) WT RD showed an organized morphology with respect to both size and arrangement. No significant changes were observed after SMGL administration (**A**(**iii**)). (**A**(**ii**)) WT HFD VAT-cells were observed to be bigger in size when compared to the WT RD cells. (**A**(**iv**)) After treatment, we observed a more uniform arrangement. (**A**(**v**)) 5xFAD RD cells were larger in size with an uneven arrangement, and a significant infiltration of macrophages that formed crown-like structures was observed. (**A**(**vii**)) Post-SMGL administration, the number of macrophages was significantly reduced, and the cell size and proper arrangement were restored. (**A**(**vi**)) Finally, the 5xFAD HFD group showed no macrophages but larger cells in diameter, which were restored after SMGL administration (**A**(**viii**)). (**B**) CD36 (red) and F4/80 (green) co-staining of visceral adipose tissue at 400× magnification. Representative sections for all groups with the distance between individual adipocytes indicated. (**B**(**i**)) WT RD showed optimal adipocyte arrangement. After SMGL administration, the cells were observed to be smaller in diameter and retain their arrangement (**B**(**iii**)). (**B**(**ii**)) WT HFD adipocytes were observed to be bigger in size when compared to the WT RD cells. (**B**(**iv**)) After treatment, we observed a more uniform arrangement and smaller size. (**B**(**v**)) 5xFAD RD cells were larger in size with an uneven arrangement. (**B**(**vii**)) Post-SMGL administration, the cell size was larger in diameter and even more irregularly arranged. (**B**(**vi**)) Finally, the 5xFAD HFD adipocytes with a larger diameter were restored after SMGL administration. There was also a more prominent signal of the co-staining, indicating the presence of macrophages. However, in (**B**(**viii**)), the arrangement deteriorated.

Histological analysis of the visceral adipose tissue (VAT) revealed distinct diet- and treatment-dependent differences in adipocyte structure across the genotypes (Figure 10A,B). Enlarged adipocytes were uniformly observed throughout the visceral fat pads of HFD-fed animals, consistent with lipid accumulation and metabolic stress (Figure 10A(i,vi)).

SMGL treatment markedly reduced the average adipocyte size in both the WT-HFD and 5xFAD-HFD groups (*p* < 0.05), providing a partial reversal of diet-induced hypertrophy (Figure 10A(iv,viii)). This reduction was more pronounced in the 5xFAD-HFD+SMGL group, where adipocytes exhibited smaller and more uniform morphology relative to their untreated HFD counterparts. In contrast, WT-HFD+SMGL mice displayed a modest but significant decrease in adipocyte diameter compared to WT-HFD mice. No significant morphological differences were observed between the RD-fed groups regardless of genotype or treatment. Notably, 5xFAD mice on RD exhibited slightly smaller adipocytes than WT-RD mice, consistent with the leaner phenotype and altered metabolic profile previously described for this model.

Overall, SMGL treatment attenuated diet-induced adipocyte hypertrophy in both WT and 5xFAD mice, with a stronger corrective effect in metabolically challenged 5xFAD-HFD mice.

### 2.10. Semaglutide Reduced Macrophage Infiltration and Lipid Transporter Expression in VAT

To further investigate adipose tissue inflammation and lipid metabolism, VAT sections were stained for the macrophage marker F4/80 (green) and lipid transporter CD36 (red) (Figure 10B). In untreated HFD-fed animals, F4/80-positive cells were frequently clustered around enlarged adipocytes, forming crown-like structures characteristic of macrophage infiltration and localized inflammatory activity [24]. This pattern was evident in both WT-HFD and 5xFAD-HFD mice, with greater signal intensity and clustering observed in 5xFAD-HFD tissues (Figure 10B(ii,iv)).

Following SMGL treatment, the F4/80 signal was visibly reduced in both the WT-HFD+SMGL and 5xFAD-HFD+SMGL groups (Figure 10B(iv,iiiv)). In RD groups, F4/80 staining remained sparse and unchanged by treatment (Figure 10B(iii,iiv)).

CD36 immunoreactivity, localized along adipocyte membranes, was elevated in HFD-fed animals compared to RD controls, consistent with increased FA uptake and lipid accumulation. SMGL treatment modestly decreased CD36 signal intensity in both WT and 5xFAD HFD-fed groups, paralleling the observed reduction in adipocyte size (Figure S4) and macrophage clustering.

## 3. Discussion

This study demonstrates that semaglutide exerts both metabolic and neuroprotective effects in a highly context-dependent manner in 5xFAD mice. Across behavioral, biochemical, and histological readouts, the strongest benefits emerged under metabolic stress, particularly in 5xFAD animals maintained on HFD. These findings are consistent with clinical and preclinical work showing that GLP-1 receptor agonists exert greater neuroprotective effects under metabolic challenge and display limited activity in metabolically intact systems [25,26]. The combined results link peripheral metabolic recovery with central improvements in amyloid pathology, inflammation, and neuronal structure, suggesting that semaglutide may affect both peripheral metabolic measures and central pathological indicators, even though this study was not designed to determine the causal mechanisms linking these effects [27].

Metabolic results provide the first indication of this coordinated response. HFD produced pronounced weight gain in both genotypes and impaired glucose tolerance, with 5xFAD mice showing a more rigid metabolic profile (Figure 1 and Figure 2), similar to previous reports of metabolic inflexibility in AD models [28,29]. SMGL selectively corrected these issues, reducing body weight, lowering glucose spikes during OGTT, and improving AUC values in 5xFAD HFD animals. Total and LDL cholesterol were significantly reduced following treatment, especially in 5xFAD HFD mice (Figure 9), and adipokine balance shifted toward a more favorable profile. This pattern is consistent with work demonstrating that semaglutide and related agents increase adiponectin, reduce leptin, and reverse diet-induced adipose inflammation [30,31].

Behavioral data mirrored these systemic corrections. Cognitive performance in the Y-maze was strongly impaired in 5xFAD mice and further reduced by HFD. Semaglutide improved alternation scores specifically in 5xFAD HFD animals, restoring performance to levels comparable to those of WT RD controls (Figure 3). The present results show that cognitive benefit occurred alongside metabolic correction, suggesting that the downstream neural effects of the GLP-1 receptor pathway may depend on the physiological context. While this test provides an indication of working memory and hippocampal function, it does not capture the full spectrum of cognitive domains. Additional low-stress behavioral paradigms would further strengthen future assessments of cognitive outcomes in this model.

Our results contrast starkly with the recent findings of Forny-Germano et al. [32], who observed no cognitive or pathological rescue in 5xFAD mice following semaglutide treatment. We propose that this difference is rooted in the metabolic context; while the aforementioned study used mice on a standard diet, our model specifically examined the ‘synergy’ between AD and a high-fat diet (HFD). This suggests that SMGL’s neuroprotective efficacy may be context-dependent, manifesting primarily when it is correcting underlying metabolic dysfunction. Furthermore, while we relied on the Y-maze to minimize the confounding effects of water-based or aversive stress in our aged, obese cohort, the robust restoration of synaptophysin and βIII-tubulin (Figure 7) provides a strong neurobiological validation for the cognitive improvements observed.

The central effects of SMGL align with our observations. Amyloid burden, assessed by immunostaining and thioflavin S, was significantly reduced in treated 5xFAD animals on either diet, with the strongest decrease observed in 5xFAD HFD mice (Figure 4). Similar reductions in Aβ deposition following GLP-1 receptor activation have been reported in both 5xFAD and APP/PS1 mice, supporting a direct or indirect modulatory role of this pathway in amyloid metabolism [33]. Soluble Aβ40 and Aβ42 also declined after treatment, matching the histological findings. These reductions coincided with restored GLP-1 receptor expression in the cortex and hippocampus (Figure 6), which is in line with studies showing that GLP 1 signaling is down regulated in AD and HFD conditions and can be restored by incretin-based therapies [34].

Neuronal and synaptic markers showed similar patterns. Levels of synaptophysin and tubulin βIII were reduced in 5xFAD mice and further diminished by HFD (Figure 7). Semaglutide improved both markers in 5xFAD HFD animals, indicating a partial rescue of synaptic and cytoskeletal integrity. Comparable improvements in synaptic proteins have been observed in GLP-1 RA-treated AD models, which report enhanced synaptic plasticity, increased mitochondrial function, and reduced oxidative stress [35]. Neuroinflammation displayed parallel improvements. Iba1 and CD68 immunoreactivity were highest in 5xFAD HFD mice and decreased substantially following treatment (Figure 8). These findings agree with work showing that GLP 1 receptor agonists reduce microglial activation, shift microglia toward a less inflammatory phenotype, and limit macrophage dependent amplification of neuroinflammation [36].

The adipose tissue findings strengthen this interpretation. HFD feeding produced marked adipocyte hypertrophy and dense macrophage infiltration in VAT, a pattern that was especially pronounced in 5xFAD mice (Figure 10). These observations are consistent with the established link between adipose inflammation and systemic metabolic dysfunction [37]. Similar reductions in adipose macrophage clustering and lipid transport abnormalities have been reported in HFD animals treated with GLP-1 receptor agonists. The organization of the adipose tissue effectively mirrors the metabolic landscape of each animal, and its close coupling with brain inflammatory signatures is consistent with an interaction between metabolic and inflammatory processes, which should be tested directly in future mechanistic studies.

A distinct and important outcome of this study was that semaglutide did not benefit metabolically normal animals. WT mice on a regular diet displayed no significant metabolic disturbances and did not improve with treatment. Instead, they showed a reproducible decline in Y-maze performance (Figure 3). This distinction reinforces the concept that glucose-lowering agents with central activity may become disruptive when homeostatic balance is intact.

The results position SMGL as a treatment whose efficacy depends strongly on the metabolic context. In 5xFAD mice under high-fat conditions, the drug corrected systemic dysfunction, reduced adipose inflammation, alleviated amyloid burden, restored GLP 1 receptor expression, improved synaptic markers, and enhanced cognition. In contrast, WT RD animals derived little or no benefit, and in some cases showed adverse cognitive effects. This contrast aligns with clinical reports indicating that GLP-1 receptor agonists yield the greatest neurological improvements in individuals with metabolic comorbidities, such as obesity, insulin resistance, or dyslipidemia [25]. These findings support a precision medicine approach in which the metabolic phenotype guides the use of GLP-1-based therapies.

In our study, several histological and behavioral analyses were conducted with relatively small sample sizes, particularly in experiments requiring extensive tissue processing and imaging-based quantification. Although statistically significant changes were detected across multiple independent readouts, smaller cohort sizes may reduce sensitivity for subtle effects and increase variability between groups.

On the other hand, the overall study involved a large number of animals that had to be distributed across eight experimental conditions incorporating genotype, dietary status, and semaglutide treatment variables. This multifactorial design was necessary to investigate the interaction between metabolic dysfunction and AD pathology under distinct metabolic states. Due to the complexity and longitudinal nature of this study, along with the extensive molecular and histological characterization performed, sample allocation differed across assays depending on tissue availability and experimental requirements.

The translational relevance of these findings becomes clearer when considered in light of human epidemiology. Metabolic disturbances frequently appear years before cognitive symptoms, and midlife obesity, dyslipidemia, and insulin resistance are among the strongest predictors of AD. Several longitudinal studies show that individuals with T2D or MetS have a substantially accelerated trajectory of cognitive decline [12,13]. Our data resonate with this real-world pattern. In 5xFAD mice, metabolic impairment created a physiological context in which amyloid accumulation, neuroinflammation, and synaptic vulnerability were amplified, and it was precisely within this state that SMGL produced its most robust benefits. Although we cannot directly conclude that treating metabolic dysfunction delays disease onset, the close correspondence between metabolic correction, reduced inflammatory load, and improved neural outcomes supports the view that early restoration of metabolic homeostasis may influence the course of neurodegenerative processes. These findings therefore align with the broader hypothesis that addressing early or co-occurring metabolic abnormalities before cognitive decline may represent a feasible strategy to delay or reduce the severity of AD, particularly in individuals with established metabolic comorbidity. However, these translational implications must be interpreted cautiously. The data are derived from a strongly amyloid-driven transgenic model subjected to high-fat-diet-induced metabolic stress, which lacks tau readouts and does not fully replicate the complexity of sporadic human AD.

## 4. Materials and Methods

### 4.1. Mice Treatment and Tissue Harvesting

5xFAD mouse models on a B6/SJL background were utilized in this study. This mouse model expresses a total of five AD-linked mutations on the human APP and PSEN1 transgenes, namely, Swedish (K670N/M671L), Florida (I716V), and London (V717I) mutations in APP, and M146L and L286V mutations in PSEN1 [18]. This model exhibits severe amyloid pathology as early as 1.5 months of age, with high levels of intraneuronal Aβ42, which form β-sheet aggregates, beginning to accumulate extracellularly at approximately 2 months of age. By 6 months, plaques are found throughout the hippocampus and cerebral cortex, extending to the thalamus, brainstem, and olfactory bulb. There are no plaques reported in the cerebellum. In this mouse model, tau tangles are also reported to be absent [38].

Hemizygous 5xFAD mice (B6SJL) were crossed with wild-type (WT) B6SJL mice, and the hemizygous offspring were collected to establish the animal treatment groups. WT mice born at the same time were also used as age- and sex-matched healthy controls in all experiments. In accordance with the ARRIVE guidelines [39], all animals were kept in a regular 12 h light/12 h dark cycle throughout the experiments and had free access to both water and food, except during fasting, which was required for the glucose tolerance tests. Our animal facility is maintained under specific pathogen-free (SPF) conditions. The study protocol was approved by the Cyprus Veterinary Authority, and all experiments involving animals were conducted in accordance with Directive 86/609/EEC, ensuring the proper treatment of animals throughout their lives and during euthanasia.

The experiments described here involved both female and male mice (n = 74). Animals were kept in separate cages depending on their sex until they were 3 months old. Following treatment initiation, the mice were further separated based on their specific treatment. Genotyping for this transgenic line was performed using PCR, as previously described [40]. Animals were randomly assigned to experimental groups prior to treatment. Owing to the longitudinal design and the requirement for explicit group identity in dietary and pharmacological interventions, full blinding during experimentation and statistical analysis was not feasible. To minimize subjective bias, all behavioral testing followed standardized protocols, quantitative image analyses were performed using automated thresholding in ImageJ (version 1.54S) with identical parameters across groups, and biochemical assays were conducted in duplicate or triplicate. Statistical analyses were performed using predefined criteria and objective endpoints.

A 60 kcal% high-fat diet (HFD) was obtained from Research Diets (New Brunswick, NJ, USA, D12492). Semaglutide (Ozempic^®^, 1 mg) was acquired from Angelidou Pharmacy (Nicosia, Cyprus) and stored at 40 °C as described by the manufacturer (Novo Nordisk, Kalundborg, Dermark). Prior to treatment, the drug was dissolved in water for injection (DEMO S.A, Krioneri, Greece) to administer 0.3 mg/kg of drug in each mouse.

The mice were placed on a regular or HFD for 5 months ad libitum before the 13-week semaglutide treatment began (once weekly, 0.3 mg/kg) intraperitoneally (IP).

Mice were euthanized by anesthetization with tribromoethanol (Avertin) via intraperitoneal (IP) injection (250 mg/kg), followed by cardiac exsanguination with 1× phosphate-buffered saline (PBS). Brain tissues were immediately harvested, and the two hemispheres were separated at the longitudinal fissure (sagittal plane). One hemisphere was exclusively used for protein quantification and immediately frozen at −80 °C without any prior processing. The other hemisphere was used solely for immunohistochemistry (IHC) and was paraffin-embedded after overnight fixation in 4% paraformaldehyde (PFA). Visceral adipose tissue (VAT) was also harvested, paraffin-embedded, and fixed overnight in 4% PFA for immunofluorescence analysis and thioflavin-S staining. The experimental design details are shown in Table 1.

Animals were assigned to experimental groups through random allocation, following stratification by genotype, diet, and sex where applicable. Behavioral testing and endpoint quantifications were conducted using coded identifiers for animals or samples whenever possible. Histological image acquisition and quantification, along with biochemical measurements, were performed with the treatment group blinded, and group identity was disclosed only after data extraction. Full blinding during treatment administration and routine animal monitoring was not feasible, as the diet/treatment conditions were known to the investigator.

### 4.2. Oral Glucose Tolerance Test (OGTT)

OGTT was performed twice during the diet and treatment, once before semaglutide treatment began and once after it ended (before euthanizing). The protocol was performed as previously described [41]. The animals were fasted for a minimum of 6 h, and a 30% glucose solution was prepared. The appropriate amount of supplemental glucose required for each mouse was calculated by multiplying the animal’s body weight by 6.667. A needle was used to make a puncture at the tip of the animal’s tail to collect a drop of blood. The first measurement was taken before any glucose administration was performed to collect the sample at t = 0. Glucose was administered by oral gavage, and a measurement was taken at a time according to the protocol following glucose delivery. A glucometer with glucose strips (On Call Plus II Blood Glucose Meter, ACON laboratories, Inc., San Diego, CA, USA) was used to measure glucose levels at five different time points (0, 15, 30, 60, and 120 min) in both WT and 5xFAD mice.

### 4.3. Y-Maze Spontaneous Alternation Test

The Y-maze is a spontaneous alternation, non-reward-based behavioral test mostly employed when evaluating short-term and working memory, both greatly related to hippocampal rigor and essentially exemplifying the level of cognitive impairment associated with the disease prior to and post-treatment. Animals are allowed to freely explore the Y-shaped maze (arms orientated at 120° from each other) for a fixed period of 6 min (n = 43). Throughout this period, the arms entered are recorded and the number of successful triads are calculated. A successful triad is counted when all four limbs of the mouse enter the maze arm. It is estimated as a triadic alternation as follows: ABC, ACB, BAC, BCA, CAB, CBA. The final value is calculated via the formula Spontaneous Alternation (%) = (Number of successful triads)/(Maximum number of triads) × 100. Lower values signify cognitive impairment.

### 4.4. Immunofluorescence, Thioflavin-S Staining, Β-Amyloid Quantification

Brain tissues embedded in paraffin, obtained from euthanized study animals, were used to obtain 5 μm sagittal sections on charged glass slides via microtome sectioning. Sections were deparaffinized and rehydrated as previously described [40]. Thioflavin-S staining was used to evaluate the presence and number of amyloid plaques in conjunction with IHC against beta-amyloid species on the same slides (n = 42). For thioflavin-S staining, a 1% solution of the stain in water was used as previously described (Fella et al.). Regarding beta-amyloid IHC, overnight incubation was performed with the B-4 primary antibody (Santa Cruz Biotechnology, sc-28365, 1:200, Paso Roblez, Santa Cruz, CA, USA), followed by 1 h of incubation with the secondary antibody (goat anti-mouse IgG [H þ L] Alexa Fluor Plus 555, Invitrogen (Carlsbad, CA, USA), A32727 1:2000) (n = 37). Other antibodies, including GFAP and CD68, were also used on other glass slides, with DAPI staining (Thermo Fisher Scientific, 62248, Waltham, MA, USA) utilized for nuclear labeling. VAT IHC was also performed to assess the presence of macrophages using anti-CD68 antibodies. Reduced sample numbers in certain histological experiments resulted from tissue availability, section quality control and the extensive processing requirements associated with immunohistochemical and imaging analyses.

A Zeiss Axionvision fluorescence microscope (Carl Zeiss Microimaging, Oberkochen, Germany) was used in collecting images of both the fibrillar amyloid plaques (green color) and the pre-fibrillar b amyloid species (red color). The software used for image capture was ZEISS ZEN 3.11 Zen 2 (blue edition). Images were obtained using 20× and 40× objective lenses (N-Achroplan and Plan-Apochromat, respectively, Jena, Germany), viewed with a Zeiss Viluma 5 illuminator. Overlapping images were collected at 5× magnification to obtain a complete view of both the cortex and hippocampus. Images were joined in Microsoft ICE (Image Composite Editor) (version 2.0.3.0) to create a single image, and the positive area was quantified using ImageJ software (Version 1.54S). The measured staining is represented as a percentage of the red or green positive surface area. Measurements were obtained from three sections per animal, and the average was used.

### 4.5. Enzyme-Linked Immunosorbent Assay (ELISA) for Aβ40/42

ELISA was performed to measure the levels of Aβ40 and Aβ42 amyloid peptides. For the ELISA experiment, 100 mg of frozen brain hemisphere was used (n = 57). Tissues were homogenized and lysed for protein extraction, as explained in detail in the kit’s manuscript (Aβ40; KMB3481, Invitrogen, and Aβ42; KMB3441, Invitrogen). Briefly, the tissue was initially homogenized by sonication in guanidine-HCl-containing lysis buffer, and the supernatant, which was collected upon centrifugation, was further diluted 10 times with 1× phosphate-buffered saline (PBS) supplemented with protease inhibitors (11836170001, Roche, Switzerland). The samples were further diluted using the standard diluent buffer and applied to the coated microplate at a final dilution of 1:10 for Aβ40 and 1:150 for Aβ42. The standards were prepared by performing serial dilutions as described in the protocol, and absorbance was obtained at 450 nm for standards and samples. Samples were used in duplicates or triplicates.

### 4.6. ELISA for Leptin, Adiponectin, Glp1r and Glut3

ELISAs were conducted to measure the levels of leptin (n = 45) (AssayGenie: MOFI00070), adiponectin (n = 51) (AssayGenie: MOFI00001), GLP1R (n = 40) (AssayGenie: MOFI01360), GLUT3 (n = 51) (AssayGenie: MOFI01396), SYP (n = 48) (AssayGenie: MOFI01130), and tubulin-β3 (n = 58) (AssayGenie: MOEB1581) in the brain, as described by the kit’s instructions. Mice brains were sonicated for homogenization in 1× PBS supplemented with protease inhibitors. The samples were further diluted using the sample diluent buffer at 1:20, 1:50, 1:10, and 1:5 dilutions for leptin, adiponectin, GLP1R, and GLUT3, respectively. The assay was performed at 370 C, and the absorbance of standards and samples was measured at 450 nm. For each ELISA, samples were analyzed in duplicate.

### 4.7. Total Cholesterol, HDL, LDL, and Triglyceride Measurements

Total cholesterol (n = 66), HDL (n = 71), LDL (n = 44), and triglyceride (TG) levels (n = 64) were measured from mouse serum. The samples were sent to the Medifos Center of Laboratory Medicine. The center has ISO-approved molecular diagnostic equipment.

### 4.8. Statistical Analysis

Statistical analyses were conducted using GraphPad Prism for Windows (version 8.00; GraphPad Software, San Diego, CA, USA). Unpaired Student’s *t*-tests and one-way and two-way ANOVAs were used to create graphical charts representing the data. Power analysis was performed using the pwr R package, which showed that the sample sizes were adequate for statistical analysis. The primary outcome measures of this study were body weight changes, OGTT/AUC measurements, and β-amyloid quantification. Histological and immunofluorescent analyses were considered secondary exploratory outcomes intended to complement the primary biochemical findings. Sample sizes were determined based on previous studies, using the 5xFAD model and practical considerations associated with long-term dietary intervention experiments.

Both male and female mice were included in the experimental cohorts. While early sex-dependent differences in amyloid pathology have been described in the 5xFAD model [38], our preliminary analyses of the data at the 9-month endpoint revealed no significant sex-specific differences in amyloid load or primary metabolic outcomes. Because the pathology had likely reached a comparable stage in both sexes at this advanced age, sex was not included as an independent variable, and the data were pooled for final analyses.

## 5. Conclusions

This study shows that semaglutide produces substantial metabolic and neuroprotective benefits in the 5xFAD model only when metabolic dysfunction is present. In HFD, SMGL corrected systemic abnormalities in glucose regulation, lipid metabolism, and adipokine signaling, and these improvements occurred alongside reductions in amyloid accumulation, microglial activation, and synaptic loss. The close alignment of metabolic restoration with central pathology indicates that the metabolic state is a determinant of therapeutic response.

WT mice on a regular diet did not benefit and displayed a modest decline in cognitive performance, suggesting that GLP-1 receptor activation may disturb neuronal balance when metabolic homeostasis is intact. This finding highlights the importance of matching metabolic interventions to the physiological context.

Overall, the results support the concept that metabolic dysfunction increases vulnerability to Alzheimer’s-related mechanisms in this model. While these preclinical findings cannot directly confirm human disease modification, they provide a strong testable hypothesis that early correction of systemic metabolic imbalance may be relevant to the course of neurodegeneration.

## Figures and Tables

**Table 1:** Experimental design. H = high-fat diet (HFD); SM = semaglutide; S = sacrifice.

Treatment	Number of Mice	Months of Age								
		1	2	3	4	5	6	7	8	9
1. H2O	WT (14: 7 ♂, 7♀)									**S**
2. H2O-SMGL	WT (13: 6♂, 7♀)					**SM**	**SM**	**SM**	**SM**	**S**
3. HFD	WT (10: 5♂, 5 ♀)			**H**	**H**	**H**	**H**	**H**	**H**	**S**
4. HFD-SMGL	WT (11: 6♂, 5 ♀)			**H**	**H**	**H, SM**	**H, SM**	**H, SM**	**H, SM**	**S**
5. H2O	5xFAD (7: 4♂, 3 ♀)									**S**
6. H2O-SMGL	5xFAD (7: 4♂, 3 ♀)					**SM**	**SM**	**SM**	**SM**	**S**
7. HFD	5xFAD (7: 4♂, 3 ♀)			**H**	**H**	**H**	**H**	**H**	**H**	**S**
8. HFD-SMGL	5xFAD (8: 4♂, 4 ♀)			**H**	**H**	**H, SM**	**H, SM**	**H, SM**	**H, SM**	**S**

## Data Availability

The original contributions presented in this study are included in the article/Supplementary Material. Further inquiries can be directed to the corresponding author.

## Supplementary Materials

The following supporting information can be downloaded at: https://www.mdpi.com/article/10.3390/ijms27125311/s1.

## Abbreviations

The following abbreviations are used in this manuscript:

AD	Alzheimer’s disease
MetS	Metabolic syndrome
SMGL	Semaglutide
RD	Regular diet
HFD	High-fat diet
LDL	Low-density lipoprotein
Aβ	Beta-amyloid
GLP1	Glucagon-like peptide-1
Iba1	Ionized calcium-binding adaptor molecule 1
CD68	Cluster of differentiation 68
CD36	Cluster of differentiation 36
WT	Wild type
T2D	Type 2 diabetes
IR	Insulin resistance
FDA	Food and Drug Administration
APP	Amyloid precursor protein
PSEN1	Presenilin 1
SPF	Specific-pathogen-free
PCR	Polymerase chain reaction
IP	Intraperitoneal
PFA	Paraformaldehyde
VAT	Visceral adipose tissue
OGTT	Oral glucose tolerance test
IgG	Immunoglobulin G
GFAP	Glial fibrillary acidic protein
ICE	Image composite editor
ELISA	Enzyme-linked immunosorbent assay
GLP1R	GLP1 receptor
GLUT3	Glucose transporter 3
HDL	High-density lipoprotein
AUC	Area under curve
Tubβ3	Tubulin beta III
SYP	Synaptophysin
