# Supplementary material for: Semaglutide Selectively Improves Metabolic and Cognitive Function in 5xFAD Mice
Source: Int J Mol Sci. 2026 Jun 11;27(12):5311. doi: 10.3390/ijms27125311 (PMC13300714; doi:10.3390/ijms27125311)

Figure S1: Monthly Animals' Weight Records

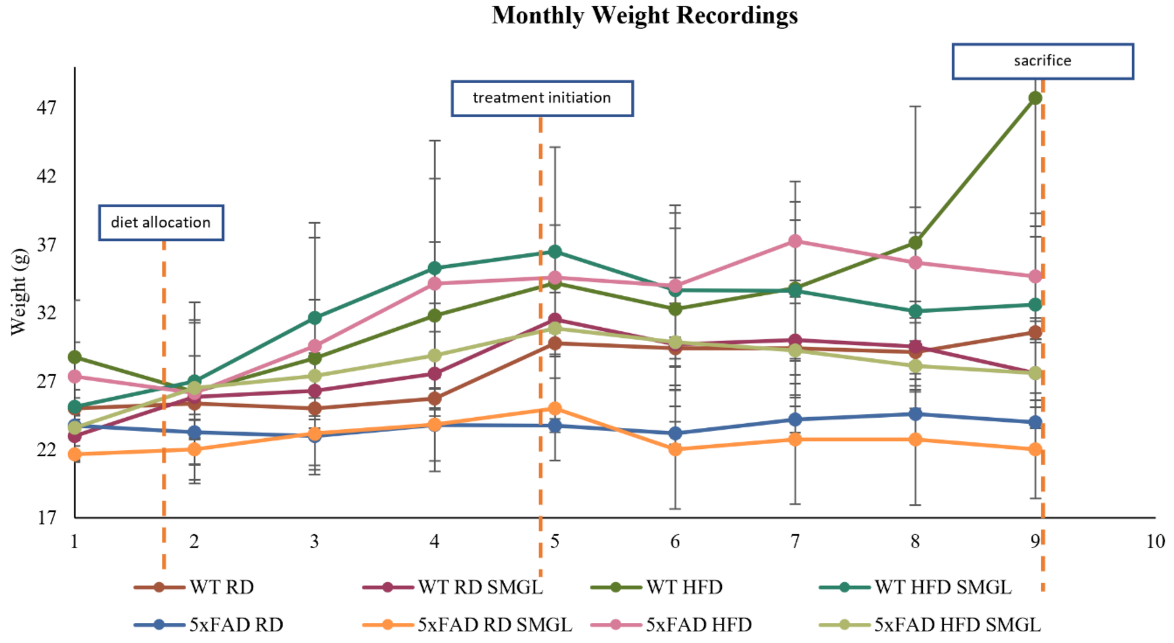

Figure S2: Individual OGTT values

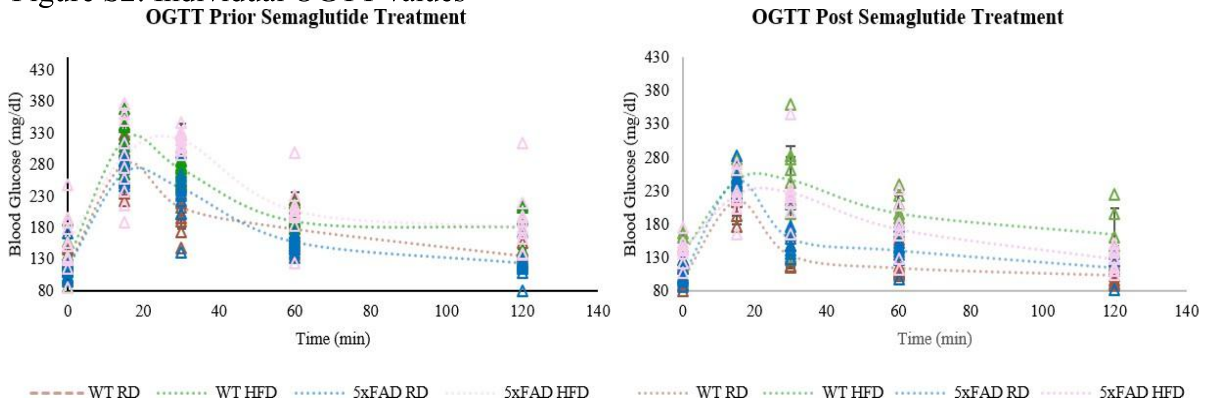

Figure S3: Representative images from WT animals stained for Thioflavin-S and Amyloid-β

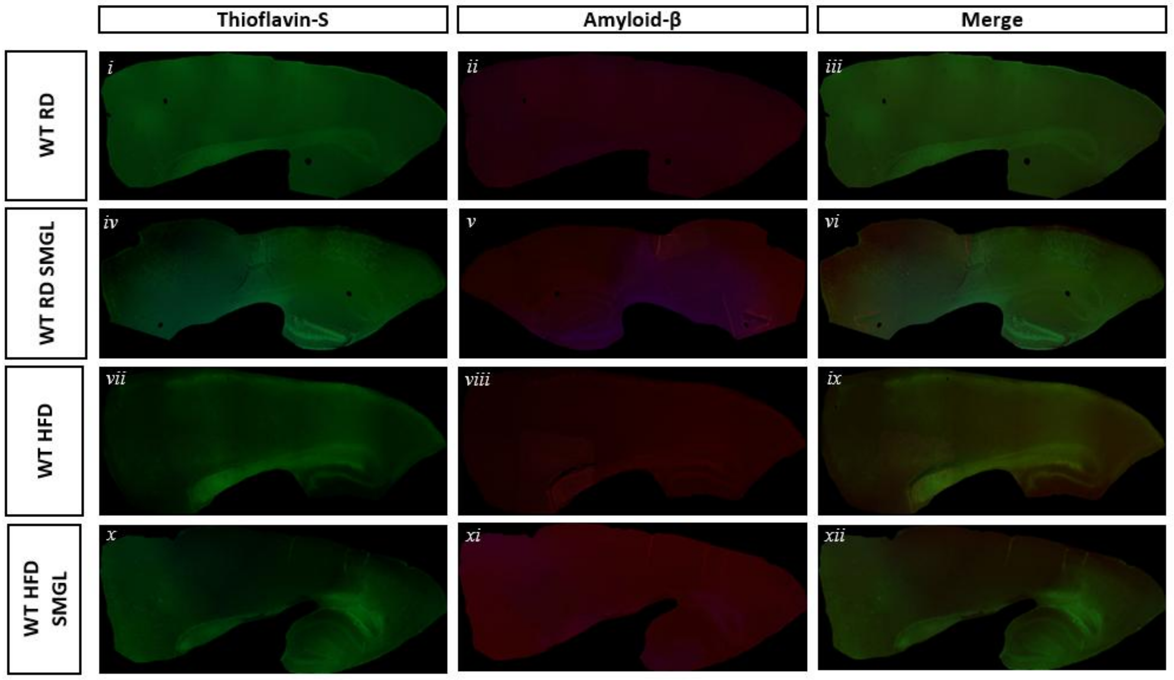

Figure S4: Average adipocyte size depending on treatment group.

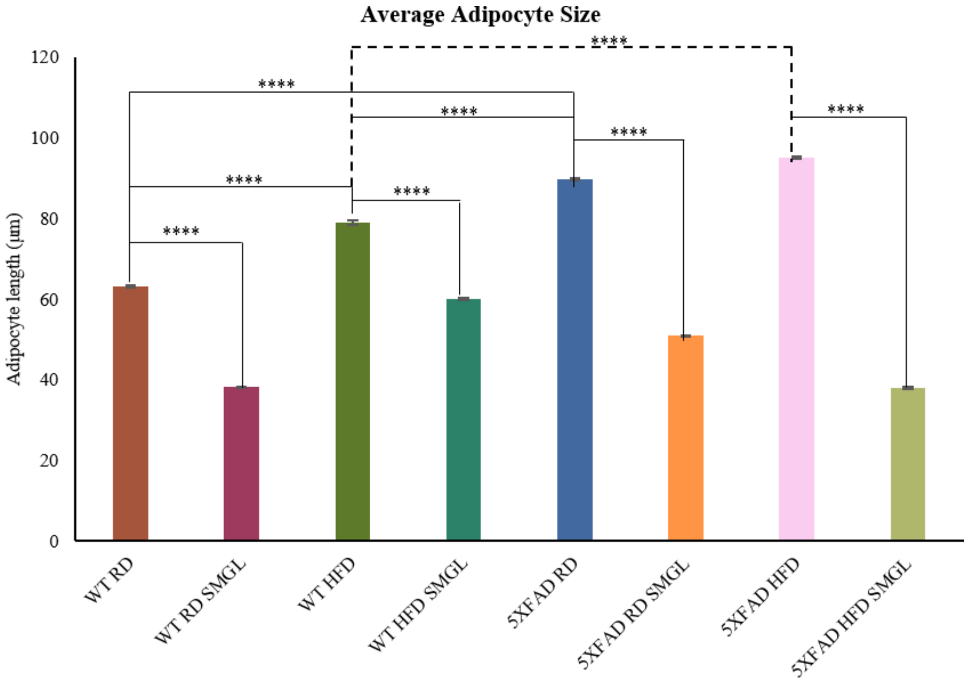

Supplement: Supplementary file 1 [file ijms-27-05311-s001.zip › ijms-4316399-supplementary.pdf]
